# Revealing the binding modes and the unbinding of 14-3-3σ proteins and inhibitors by computational methods

**DOI:** 10.1038/srep16481

**Published:** 2015-11-16

**Authors:** Guodong Hu, Zanxia Cao, Shicai Xu, Wei Wang, Jihua Wang

**Affiliations:** 1 Shandong Provincial Key Laboratory of Functional Macromolecular Biophysics and College of Physics and Electronic Information, Dezhou University, Dezhou, 253023, China; 2 National Laboratory of Solid State Microstructure and Department of Physics, Nanjing University, Nanjing, 210093, China

## Abstract

The 14-3-3σ proteins are a family of ubiquitous conserved eukaryotic regulatory molecules involved in the regulation of mitogenic signal transduction, apoptotic cell death, and cell cycle control. A lot of small-molecule inhibitors have been identified for 14-3-3 protein-protein interactions (PPIs). In this work, we carried out molecular dynamics (MD) simulations combined with molecular mechanics generalized Born surface area (MM-GBSA) method to study the binding mechanism between a 14-3-3σ protein and its eight inhibitors. The ranking order of our calculated binding free energies is in agreement with the experimental results. We found that the binding free energies are mainly from interactions between the phosphate group of the inhibitors and the hydrophilic residues. To improve the binding free energy of Rx group, we designed the inhibitor R9 with group R9 = 4-hydroxypheny. However, we also found that the binding free energy of inhibitor R9 is smaller than that of inhibitor R1. By further using the steer molecular dynamics (SMD) simulations, we identified a new hydrogen bond between the inhibitor R8 and residue Arg64 in the pulling paths. The information obtained from this study may be valuable for future rational design of novel inhibitors, and provide better structural understanding of inhibitor binding to 14-3-3σ proteins.

Protein-protein interactions (PPIs) are important features for biological processes, and alterations in PPIs events could cause diseases such as cancer and diabetes^1^,^2^. Different proteins may have different interactions between each other^3^. A specific kind of PPIs describes that a protein can interact with parts of other proteins, peptides or small molecules which are termed as the inhibitors of the protein. This protein usually plays a role of the drug target. A rich source of potential drug targets offer attractive opportunities for therapeutic intervention by addressing of PPIs with small, drug-like molecules.

The 14-3-3 proteins are a family of ubiquitous conserved eukaryotic regulatory molecules involved in the regulation of mitogenic signal transduction, apoptotic cell death, and cell cycle control^4^. This protein family consists of seven distinct isoforms in human cells (β, ϵ, γ, η, σ, τ and ζ) as well as a variety of post-translationally modified forms^5^,^6^. The 14-3-3 proteins have the ability to bind a multitude of functionally diverse signaling proteins, including kinases, phosphatases, and transmembrane receptors. They mediate their physiological effects by binding to other proteins, modulating their (clients’) subcellular localization, enzymatic activity, or their ability to interact with further proteins^7^. For example, the σ isoform has been implicated in breast cancer^8^ and is necessary for proper G_2_ checkpoint function^9^. As one of the most important “hub” proteins with at least 200–300 interaction partners, the 14-3-3 proteins are an especially fruitful case for PPI intervention^10^.

Each 14-3-3 proteins consists of characteristic cup-like shape functional dimers with each monomer has nine antiparallel α-helices displaying a so-called amphipathic groove that accommodates the mostly phosphorylated interaction motifs of their partner proteins (see Fig. 1A)^11^,^12^. Small-molecule regulation on PPIs is one of the most exciting but also difficult fields in drug development and chemical biology^13^.

Previously, several attempts have been made to develop small-molecule inhibitors for the 14-3-3 PPIs. For example, Wu *et al.* designed and synthesized a peptide-small-molecule hybrid library based on the original optimal 14-3-3 binding peptide and maintained the central phosphoserine residue^14^,^15^. Corradi *et al.* employed an *in silico* structure-based inhibitor design approach to identify the first non-peptidic small molecule compounds with anti-proliferative activity^16^. Zhao *et al.* identified and experimentally confirmed a pyridoxal-phosphate derivative, which create a covalent linkage of the pyridoxal-phosphate moiety to the residue Lys120 in the binding groove of the 14-3-3 protein^17^,^18^. Bier *et al.* reported a molecular tweezers which bind to a 14-3-3 adapter protein and modulate its interaction with the partner proteins^19^. Thiel *et al.* identified noncovalent and non-peptideic small-molecule inhibitors for extracellular 14-3-3 PPIs by virtual screening^20^.

In the work by Thiel *et al.*, the crystallographic structures of the 14-3-3σ protein and inhibitors complexes were solved. Such high-quality structural data can be exploited to design the PPI inhibitors *in silico*^20^. This is very important for the understanding the protein-inhibitor interactions at the atomic level of this class of compounds, which may lead to the development of 14-3-3σ inhibitors with better potency.

It is well-known that molecular dynamics (MD) simulations can enhance our understanding of binding mechanisms for protein-inhibitor complexes, such as the 14-3-3σ protein and its inhibitors complexes, by providing quantitative binding affinities^21^,^22^,^23^,^24^,^25^,^26^,^27^. Several computational methods with various levels of computational expense and accuracy can be used to estimate the inhibitor binding affinities and selectivities. These methods include the thermodynamic integration (TI), the free energy perturbation (FEP) method^28^,^29^, and molecular mechanics generalized Born surface area (MM-GBSA) method^30^,^31^. Among them, MM-GBSA method is a versatile tool for calculating the binding free energy of a given protein-inhibitor complex. In this method, the gas-phase energy, calculated using conventional molecular mechanics force fields such as AMBER^32^, is combined with a continuum model of solvation that includes a surface area based nonpolar contribution^33^ and a polar solvation free energy calculated with the generalized Born (GB) approximate model of electrostatics^34^. Noted that MM-GBSA method utilizes a fully pairwise potential to decompose the total binding free energy into atomic/group contributions in a structurally nonperturbing formalism^30^.

Steered molecular dynamics (SMD) simulation takes inspiration from single-molecule pulling experiments^35^, and dissociates a complex structure by a pulling force^36^,^37^. The non-equilibrium dynamics of the system under a pulling force can map out the free-energy landscape in terms of the potential of mean force (PMF)^38^ with high precision and efficiency^39^,^40^,^41^,^42^. The free-energy difference between the bound states and the dissociate states can be extracted by measuring the work along the transition paths. Thus, SMD simulations have become widely used in studying biochemical processes including the unfoulding/foulding mechanism of proteins^43^, transportation of ions and organic molecules across membrane channels^39^,^44^,^45^,^46^,^47^, and the mechanisms of protein-inhibitor binding^36^,^40^,^48^.

In this paper, we combined the MD simulation with MM-GBSA method to calculate the binding free energies between the 14-3-3σ and its eight inhibitors (Fig. 1B). Our calculated binding free energies are in agreement with the experimental results. The interaction between the phosphate group of inhibitors and the hydrophilic residues are the main contribution for the binding free energies in all compounds (14-3-3σ proteins and inhibitors). To explore the unbinding mechanism for 14-3-3σ and its inhibitors, SMD simulations combined with Brownian-dynamics fluctuation-dissipation theorem (BD-FDT) were used to calculate the interaction energy landscape of 14-3-3σ with inhibitors R1 and R8. Base on the binding model of 14-3-3σ and its inhibitors, a new inhibitor R9, which can form a new hydrogen bond between group R9 = 4-hydroxypheny and residue Glu57, was designed and evaluated in this work.

## Results

### The protonation of the phosphate group

The crystallographic complex of the phosphate peptide and the 14-3-3σ protein (PDB ID: 1YWT)^49^,^50^ revealed that the phosphate group of the binding peptide forms several hydrogen bonds with 14-3-3σ protein. The structure-based net charges at neutral pH for the 14-3-3σ protein were calculated by using the Adaptive Poisson-Boltzmann Solver (APBS) and PDB2PQ program^51^ and visualized resulting electrostatic potentials in VMD software^52^ (Fig. 2A). It is clear that the groove in 14-3-3σ protein is hydrophilic^53^. The hydrophilic pocket of the phosphate group is formed by several hydrophilic residues (Arg60, Arg133, Tyr134 and so on). In our previous work, the phosphate group in phosphoserine residue was in unprotonated state^50^. So we set the phosphate group of inhibitors in unprotonated state in this work. To evaluate the validity of unprotonated phosphate group of inhibitors, we calculated five averaged distances between the atoms of protein and the atoms of the phosphate group based on the MD trajectory from 15 ns to 20 ns in compound R1. As shown in Fig. 2B, the calculated values are in good agreement with the crystallographic values.

### Stability of the compounds

**Figure 1:**
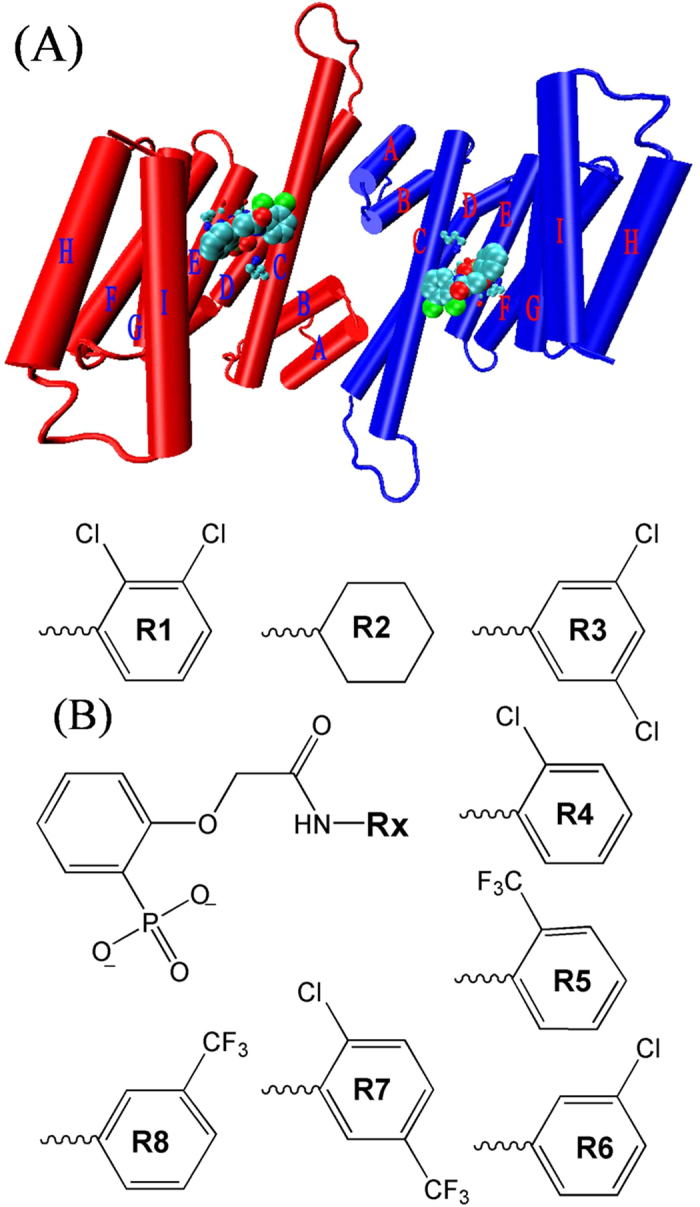
(**A**) Initial structure of the 14-3-3σ protein and its inhibitors. The two identical chains of the dimer are shown in red and blue color, respectively. Helices are shown as labeled cylinders. The inhibitors are shown in large ball representation. The key residues are shown in ball and stick representation. (**B**) Molecular structures of eight inhibitors of the 14-3-3σ protein.

MD simulations for eight compounds were performed for the time duration of 20 ns. The root mean square deviations (RMSDs) from the crystallographic structure, which can effectively assess the dynamics stability of compounds, were analyzed by using Ptraj^54^ module of AmberTools software for apo-14-3-3σ, as well as for compounds R1 and R8 (see Fig. 3). The average RMSDs of binding pocket in the last 5 ns MD simulations for apo-14-3-3σ (1.62 ± 0.16 Å) is larger than that for compound R1(1.17 ± 0.14 Å), as well as that for compound R8 (1.15 ± 0.13 Å). This indicates that the binding pocket of the 14-3-3σ protein is more stable with inhibitor than that without inhibitor. It is noted that the RMSDs for the inhibitors show large fluctuation (Fig. 3), indicating some groups of the inhibitor would not bound tightly to the proteins. To evaluate which part of the inhibitor fluctuate largely, we extracted two groups (group one: 2-hydroxyphenylphosphonic acid; and group Rxs: which names are shown in Fig. 1B) of inhibitors to calculate their RMSDs. The standard deviations of the RMSD for the inhibitors (0.33 Å and 0.55 Å) are larger than those for the group one (0.18 Å and 0.27 Å) and smaller than those for the group Rxs (0.52 Å and 0.74 Å) for compounds R1 and R8, respectively, as well as for compounds R2-R7.

### Analysis of binding free energy

We noted that 14-3-3σ would undergo conformational change caused by the binding to inhibitor. However, since we are more concerned with the ranking of the calculated binding free energies for all inhibitors with the same chemical scaffold (Fig. 1B), all the snapshots used in the MM-GBSA were extracted from the trajectories of the compounds. The binding free energies for all eight systems were calculated by using mm_pbsa program in AMBER 12 and summarized in Table 1. Though the predicted absolute free energies were larger than those of the experimental results, the ranking orders of them were in good agreement. Figure 4 shows how well the predicted free energies reproduce the experimental data. The correlation coefficient 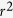 is 0.93. Besides ranking order of the binding free energies correctly, MM-GBSA method can decompose the total binding free energy into individual components, thereby enabling us to understand the complex binding process in detail^31^. For the eight compounds, the van der Waals interactions and the nonpolar solvation energies, which are responsible for the burial of inhibitor’s hydrophobic groups upon binding, are favorable for binding free energies. The mean value of the sum of van der Waals and hydrophobic interaction energies (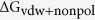) is −15.65 kcal/mol with an root-mean-square deviation of 2.79 kcal/mol. For the electrostatic energy (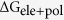), the mean value is −25.53 kcal/mol with an root-mean-square deviation of 6.51 kcal/mol. The mean value of entropic contribution (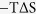) is 20.31 kcal/mol with a root-mean-square deviation of 0.78 kcal/mol. The correlation coefficients of the three energy terms (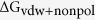, 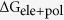, and 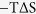) with the binding free energies are 0.30, 0.92, and 0.83 in sequence. Thus it is important to add both the electrostatic and entropic contributions for the designing of potentially new inhibitor.

### Identification of the key residues responsible for the binding of inhibitor

In order to find which residues make significant intermolecular interaction contributions to the binding with the inhibitors, the decomposition of the electrostatic interaction energy, van der Waals energy, and solvation free energy for all compounds were analyzed and the results are depicted in Fig. 5 for compounds R1 and R8 and in Fig. S1 for compounds R2-R7, respectively. The decomposition method with MM-GBSA can naturally be used for the energy decomposition at the atomic level for the per-atom contributions summed over all atoms of each residue to obtain the contribution of each residue. This has been successfully applied to a lot of protein-inhibitor binding systems. The major favorable energy contributions originate predominantly from seven residues (Lys53, Arg60, Lys126, Arg133, Tyr134, Leu178, and Val182) with averaged energy contribution larger than −0.5 kcal/mol in all compounds. Special attention had been paid to three residues (Arg60, Arg133 and Tyr134) with large electrostatic contribution. For example, the electrostatic contributions of residues Arg60, Arg133 and Tyr134 are −17.37, −19.04, and −5.0 kcal/mol for compound R1, respectively. The phosphate group has negative charge and residue arginine has positive charge, resulting in strong electrostatic attraction between them. The hydrogen bonds between the phosphate group and the 14-3-3σ protein were listed in Table 2, showing the occupancies and distances of hydrogen bonds in all compounds. The phosphate group forms three hydrogen bonds with both residues Arg60 and Arg133, as well as one hydrogen bond with residue Tyr134. Most of the hydrogen bonds are stable with high occupancy and similar distance in all compounds (Table 2), implying that the phosphate groups were tightly bonded in the binding pocked formed by three hydrophilic residues (Arg60, Arg133 and Tyr134). This result is in accordance with the analysis of RMSDs.

**Figure 2:**
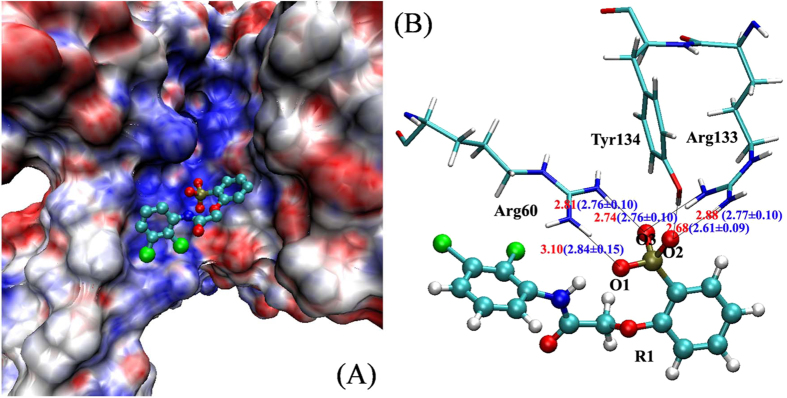
(**A**) Structure-based electrostatic potentials at neutral pH for the 14-3-3σ protein shown in surface representation. The inhibitor R1 is shown in ball and stick representation. (**B**) The distances between the oxygen atoms of the phosphate group of inhibitor R1 and the atoms of side chain of residues in the binding pocket of the phosphate group in crystallographic structure (shown in red color). Their average distances in the last 5 ns MD structures are shown in blue color.

**Figure 3:**
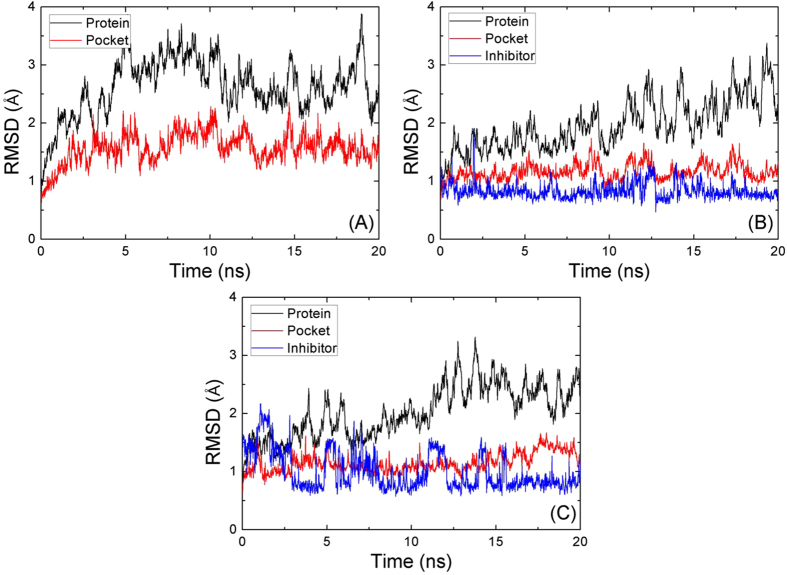
RMSDs of the backbone atoms of the 14-3-3σ protein, heavy atoms of the binding pocket (within 5 Å), and the heavy atoms in the inhibitors as a function of the MD simulation time for: (**A**) the 14-3-3σ protein without the inhibitor, (**B**) compound R1, and (**C**) compound R8 as a function of the MD simulation time.

**Figure 4:**
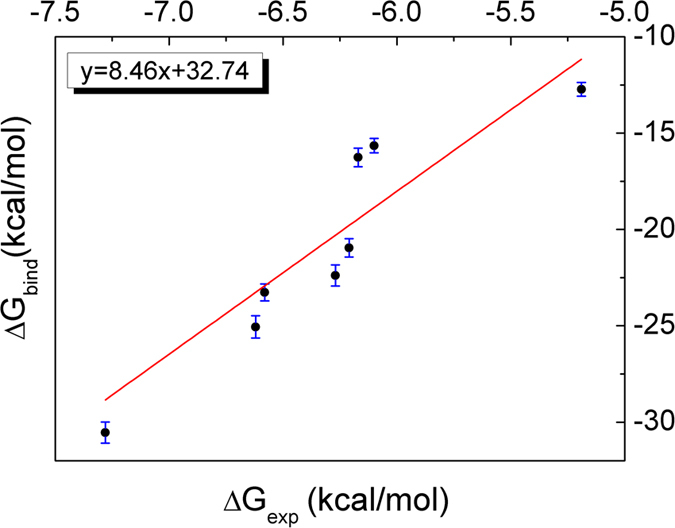
Comparison between the calculated (ΔG_bind_) and the experimental (ΔG_exp_) binding free energies.

**Figure 5:**
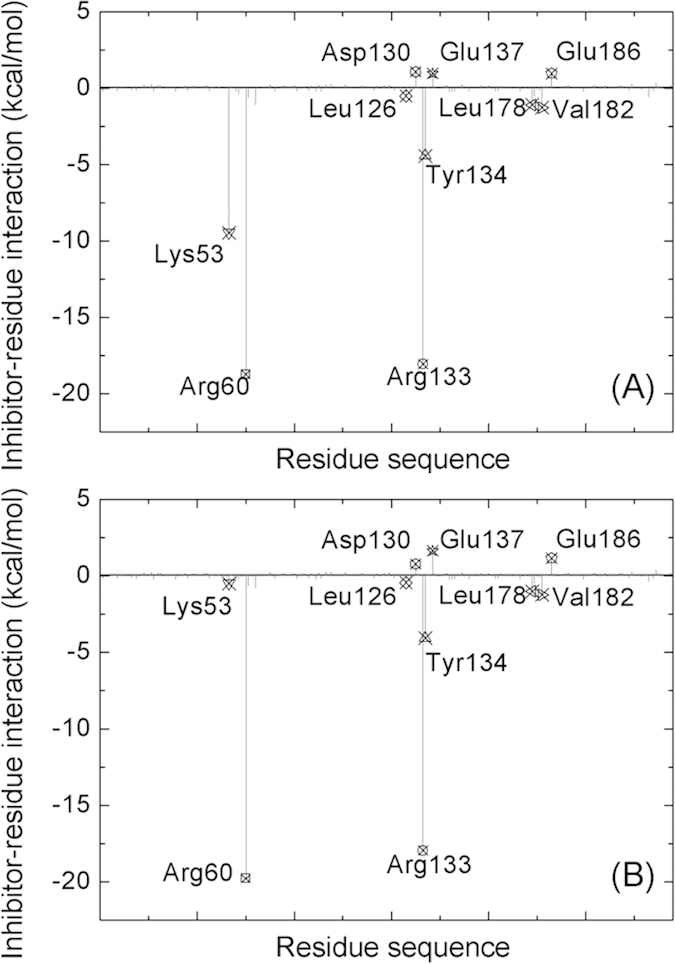
The decomposition of inhibitors on a per-residue basis for compounds R1 (**A**) and R8 (**B**).

**Figure 6:**
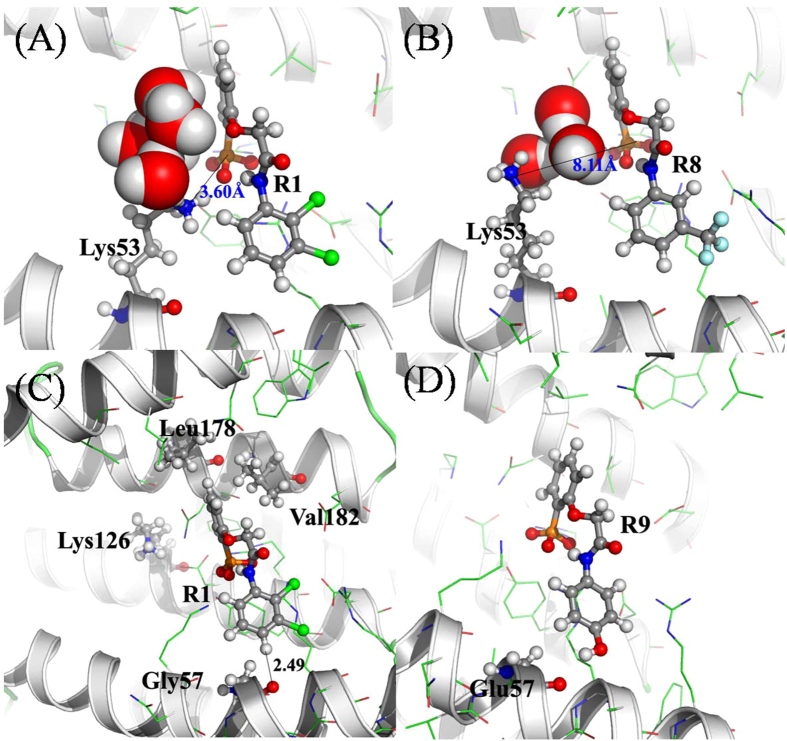
Relative positions of residue Lys53, inhibitors and the water molecules near both the residue Lys53 and inhibitor in compounds R1 (**A**) and R8 (**B**), respectively. (**C**) Four key residues and inhibitor R1. (**D**) The new designed inhibitor R9 and Glu57. The 14-3-3σ proteins are shown in ribbon, residues and inhibitors are shown in stick and ball representation, as well as water molecules in large spheres.

The side chain of residue Lys53 is in the binding pocket of the phosphate group and may contribute large electrostatic interaction energy. However, the total contributions for Lys53 in compounds R5, R6, R7, and R8 are less than 1.0 kcal/mol, which are less than those in compounds R1, R2, R3, and R4. Although the gas-phase electrostatic interaction of Lys53 is stronger in compounds R5, R6, R7, and R8, it is compensated by the polar solvation energy. We calculated the averaged distances between the nitrogen atom of side chain of Lys53 and the phosphorus atom of the phosphate group over the last 5 ns MD trajectories. By contrast, the averaged distances are smaller in compounds R1 (3.60 Å), R2 (3.57 Å), R3 (3.85 Å), and R4 (5.21 Å) than those in compounds R5 (7.66 Å), R6 (6.30 Å), R7 (6.13 Å) and R8 (8.11 Å). In order to understand the local structural features between the residue Lys53 and inhibitors in compounds R1 and R8, their relative position are shown in Fig. 6A,B, respectively. It is clearly seen from Fig. 6B that there are a few water molecules between the phosphate group of R8 and the side chain of Lys53. As shown in Fig. 6C, there are strong interactions between three residues (Lys126, Leu178, and Val182) and group one of R1, as the van der Waals energies are favorable the binding for residues Leu178 and Val182 to group one, while the electrostatic energies for Leu126. There is a π-alkyl interaction (−0.69 kcal/mol) between the side chain of Val182 and the group one of inhibitor R1. There are three unfavorable residues (Asp130, Glu137, and Glu186) for inhibitor binding to protein. The averaged free energies for these three residues in eight compounds are 0.93, 1.03, and 0.97 kcal/mol, respectively. These free energies also attributed to the electrostatic interaction. Since the residues aspartic acid and glutamic acid have negative charges, they repel the phosphate group and attract the residues with positive charge in the binding pocket. It is clear that the key residues mainly interact with the group one of the inhibitors, resulting in the formation of a pocket surrounding the group one (Fig. 2A). This is in agreement with the state that the phosphate has the strongest pharmacophoric properties^20^. By contrast, the Rxs are surrounded by several residues, while there is no stronger interaction between Rxs and residues (Fig. 5 and Fig. S1).

### SMD simulation combined with BD-FDT

**Figure 7:**
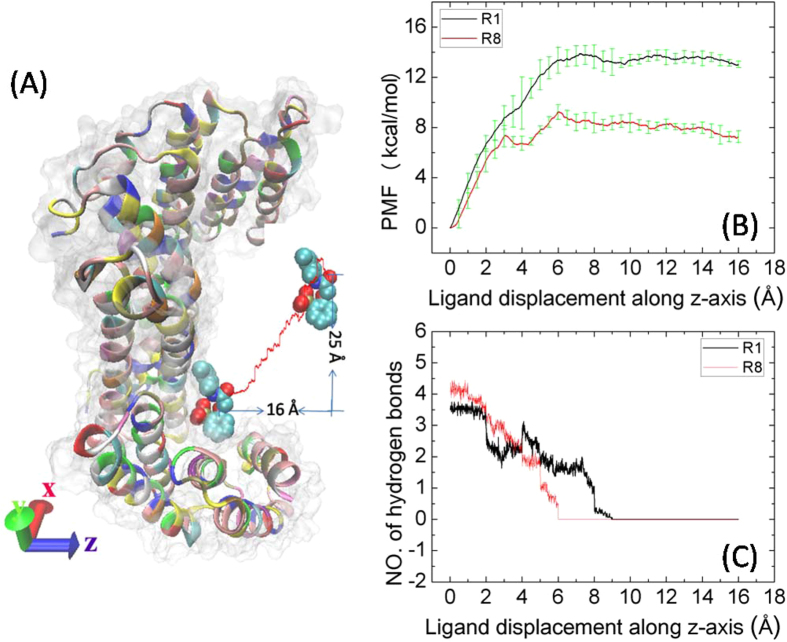
(**A**) Pulling compound R1 from its bound state to dissociated state. The 14-3-3σ protein is shown in a cartoon and a surface representation; Inhibitor R1 is shown in a ball representation. The pulling path is shown in red line. (**B**) PMFs as a function of the inhibitor displacement from its binding site along the pulling path. (**C**) The averaged number of hydrogen bonds formed between the 14-3-3σ protein and its inhibitor as a function of the inhibitor displacement.

SMD simulations were performed to investigate the dynamic processes of two inhibitors (say R1 and R8) unbinding from the 14-3-3σ protein. The starting structures of compounds R1 and R8 for SMD simulations were extracted from the last structure of the afore-presented MD simulations. Then the starting structures were rotated for the orifice of the inhibitor binding pocket toward the +z direction, put them in a box of water, and neutralized the systems. Then 10 ns equilibrated MD simulation was carried out for each system. In our SMD simulations, each inhibitor is represented by two centers. Both centers were steered at the same time along z direction. The pulling speed was set at 0.01 Å/ps in z direction. In order to reduce the impact of pulling on the 14-3-3σ protein, the inhibitor can move freely in x and y directions, and the whole pulling path was divided into 16 segments along the z-direction with 1 Å for each segment. One pulling path way of compound R1 was show in Fig. 7A, the displacement is 16 Å from the bound state to the dissociated state, as well as 25 Å in the xy plane. For each segment, two types of SMD simulations were performed: one for pulling back to (denoted as reverse) the binding site and one for pulling away (denoted as forward) from the binding site. We sampled four forward and reverse pulling paths during which the work done to the system was recorded for each segment. The curves of works done to the systems along the pulling paths are shown in Fig. S2. From these works, we calculated the PMFs as a function of the displacement of inhibitors along z-axis by using the BD-FDT and the results are shown in Fig. 7B. We can see that the PMF difference between the bound state to the dissociated state are −13.88 and −9.24 kcal/mol for compounds R1 and R8, respectively. For compound R1, the PMF rises all the way until the displacement reaches to 7 Å where the inhibitor is steered out of the binding pocket. After that, the PMF reaches a plateau. For compound R8, the PMF rises with the displacement <3 Å and reaches an interesting intermediate state around the displacement within 3.5 Å to 4.5 Å. After that, the PMF rises again and then levels off after 6 Å, indicating the inhibitor is in the dissociate state.

## Discussion

Our analysis based on RMSDs shows that the group one is more stable than the group Rxs in each compound. This result indicated that the group one bound tightly to the 14-3-3σ protein, and the group Rxs may not. Our analysis on the binding free energies shows that the electrostatic contribution plays important role in the binding of the inhibitor and the 14-3-3σ protein because the group one of inhibitors has negative charge and the bottom of the binding pocket is hydrophilic. The hydrophilic residues (Arg60, Arg133 and Tyr134) at the bottom of the binding pocket formed seven stable hydrogen bonds with the phosphate group and contributed large electrostatic energy. Additional, two residues (Leu178 and Val182) at the binding pocket of group one contribute large van der Waals energies and the residue Leu126 with large electrostatic energies. So the group one is stable in these compounds. The entropic contribution also plays important role, this is in accordance with the large RMSDs fluctuation of the group Rxs.

**Figure 8:**
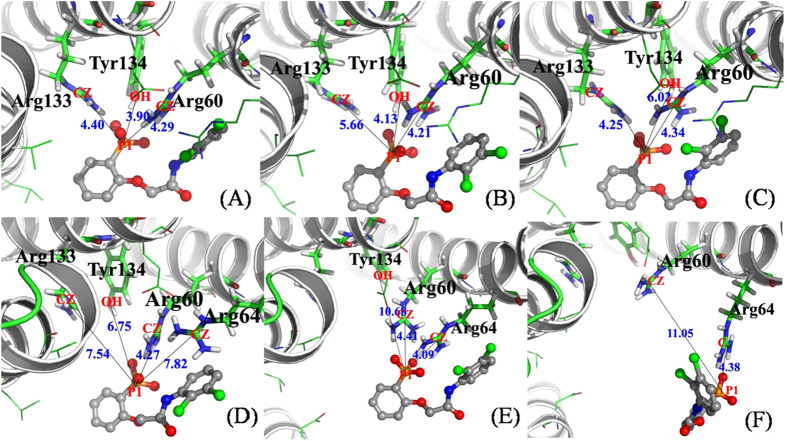
Schematic view of the key distances between the inhibitor R1 and the 14-3-3σ protein. The 14-3-3σ protein are shown in new cartoon representation, and the inhibitors are shown in stick representation and colored by name of atom. Plots (**A,B,E** and **F**) show the conformations for the center of inhibitor R1 at 1 Å, 3.5 Å, 4.0 Å, and 7.0 Å, respectively. Plots (**C**,**D)** show the conformations from different SMD trajectories for the center of inhibitor R1 at 2.5 Å. The distances, which can be used to characterize the hydrogen bond, are shown with line in black color.

As the afore-presented results, the hydrophilic interactions play important role in the inhibitor binding. So we calculated the number of hydrogen bonds between the inhibitor and the 14-3-3σ protein from our SMD simulations (shown in Fig. 7C). The number of hydrogen bonds decreases gradually along all the way until 6 Å for compound R8, indicating that there is no new hydrogen bond formed during the SMD process. For compound R1, the number of hydrogen bonds levels off during the first 2 Å of the displacement, showing stability of the hydrogen bonds formed in the starting structure of SMD simulation (Fig. 8A). From 2 Å to 4 Å, an obvious decrease indicates that some hydrogen bonds were broken, which have shown three styles for four forward and reverse SMD simulations. The broken hydrogen bonds were found for the phosphate group with either the residue Arg133 (Fig. 8B) or the residue Tyr134 (Fig. 8C), as well as both (Fig. 8D). After that, the number of hydrogen bonds increases obviously, indicating that there is a new hydrogen bond formed, then gradually decreases to zero at 9 Å. As shown in Fig. 8E, the new hydrogen bond was formed between the phosphate group and the residue Arg64. As the inhibitor was pulled away from the binding pocket, the last hydrogen bond formed in the starting structure was also broken (Fig. 8F).

Hydrogen bonds is important contributor to the specificity of receptor inhibitor interactions. As the bottom of the binding pocket around the group Rx are hydrophilic, it would be possible to form hydrogen bond between the group Rx of the inhibitor and the protein (Fig. 2A). The distance between hydrogen atom of the group Rx and the oxygen atom of backbone of residue Gly57 is 2.49 Å (Fig. 6C). So we can design an inhibitor termed as R9 with group R9 = 4-hydroxyphenyl. The same study for compound R9 were done as for compounds R1-R8. A hydrogen bond, which is our objective to form in compound R9, was observed between resiude Gly57 and the hydroxy of 4-hydroxyphenyl with occupancy of 68.4% and averaged distance of 2.92 Å. The free energy decomposition indicated that the contribution of residue Gly57 is −0.68 kcal/mol, which is the largest value among all compounds. However, the total binding free energy for compound R9 is −26.01 kcal/mol, which is smaller than that for compound R1 and larger than those for compounds R2-R8. The hydrogen bond analysis shows that most of the hydrogen bonds of the phosphate group in compound R9 are weaker than those in compound R1. The free energy decomposition also demonstrated that the binding free energies of key residues especially for Arg60 and Arg133 are smaller in compound R9 than those in compound R1. The added hydrogen bond in compound R9 increases the interaction energy of group R9 and decreases the interaction energy of group one.

In conclusion, the phosphate group of the inhibitor is in unprotonated state. The ranking order of the calculated free energy by MM-GBSA method is in agreement with the experimental data. We found that the phosphate group of the inhibitor forms strong hydrogen bonds with three residues (Arg60, Arg133 and Tyr134). The group one is more stable than group Rx in each compound. The electrostatic and entropic contributions are important for the 14-3-3σ protein binding to the inhibitors. We designed a new inhibitor R9. Although an additional hydrogen bond (group R9 and Gly57) was found in compound R9, its binding free energy is only ranked the second among all the inhibitors. We performed two pulling experiments, and found that the residue Arg64 is important in the unbinding paths of compound R8, because this residue forms a new hydrogen bond with the inhibitor R8 in the pulling paths. As a result, our study can theoretically provide dynamics information and guidance for the design of new potent inhibitors targeting the 14-3-3σ protein.

## Materials and Methods

### System setups

The crystal structures of all compounds determined by Christian Ottmann *et al.* were used as the starting structures in our MD simulations^20^. Missing loops were obtained from the crystal structure of 14-3-3σ (PDB ID: 3MHR)^55^. All crystallographic water molecules were retained in the starting model. The standard AMBER force field (FF03)^56^ was used to describe the protein parameters and water molecules. Single-point calculations with Gaussion 03 were performed to obtain the electrostatic potential around each compound by using Hartree-Fock/6-31 G* basis set^57^. Atomic partial charges of the inhibitors were fitted to the electrostatic potential (ESP) in this study by using the RESP method with the Antechamber module of AMBER12 package^58^. All compounds were solvated in a rectangular periodic box of TIP3P^59^ water molecules with a margin distance of 12 Å, and the systems were neutralized by adding an appropriate number of sodions.

### Molecular dynamics simulation

For all systems, energy minimizations and MD simulations were carried out using the AMBER12 package^58^. The solvated models were first minimized in constant volume by 1000 cycles of steepest descent minimization followed by 1000 cycles of conjugated gradient minimization. After energy minimization, the harmonic restraints with force constants of 2 kcal/(mol·Å^2^) were applied to all atoms of compound and constant volume was carried out for 70 ps, during which the systems were heated from 0 K to 300 K. Subsequent a constant-pressure MD was used for 90 ps to adjust the solvent density. Finally, a 20 ns production run of constant-pressure MD simulation was carried out at 300 K without any restraints. The MD simulations were performed with periodic boundary conditions at 300 K with the Langevin thermostat and long-range electrostatic interactions with particle mesh Ewald method^60^. All the covalent bonds involving hydrogen atoms were constrained by applying the SHAKE algorithm^61^. The time step of all MD simulations was set to be 2 fs with a cutoff of 12 Å. The pressure was kept at 1.0 atm using isotropic positional scaling. The intermediate structures were saved at every 1 ps for analysis.

### Free energy calculations

The stable MD trajectory obtained for each compound was used to estimate the binding free energies (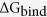) by using MM-GBSA method, which has been implemented in AMBER12 program. A total number of 500 snapshots were chosen evenly from the last 5 ns MD trajectories with an interval of 10 ps. All counterions were stripped, as well as all water molecules. Our MM-GBSA calculations for each snapshot were carried out in the same way as in other protein-inhibitor systems. Briefly, the MM-GBSA method can be conceptually summarized by the following equations:


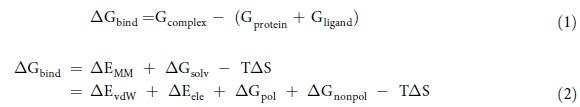


where 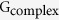, 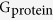 and 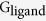 are the free energies of the complex, receptor and inhibitor, respectively. The binding free energy (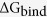) is evaluated by a sum of the changes in the molecular mechanical (MM) gas-phase binding energy (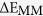), the solvation free energy (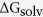) and entropic (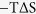) contribution. 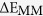 is further divided into a van der Waals (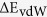) and a gas-phase electrostatic energies (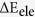). These energies were computed using the same parameter set as that used in the MD simulations. And the solvation free energy (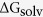) is further divided into a polar (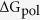) and a nonpolar (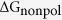) component. The polar component (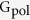) was calculated with a GBSA module of the AMBER12 suite. The nonpolar component (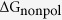) was determined using 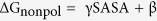, where SASA is the solvent-accessible surface area that was determined with MSMS program^62^ with a probe radius of 1.4 Å, 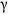 and 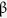 were set to be 0.005 kcl·mol^−1^·Å^−1^ and 0.0 kcal/mol, respectively^63^. The conformational entropy contributions to the binding free energies were estimated for 125 snapshots using the normal-mode analysis with AMBER NMODE module^64^.

### Steered molecular dynamics (SMD) simulations

The MD simulations and SMD simulations were performed by using NAMD 2.8 package^65^. We employed periodic boundary conditions in all three dimensions and utilized particle mesh Ewald (PME) method for treating long-range electrostatic interactions. The cut-off distance for long-range interactions was set to 12 Å with as witching distance of 10 Å. The time step was 2 fs for short-range and 4 fs for long-range interactions. The covalent bonds of all hydrogen atoms were fixed to their equilibrium length by SHAKE algorithm^61^. The temperature was maintained at 300 K using Langevin thermost at with the damping constant chosen as 5/ps and pressure at 1 bar. In our SMD, a inhibitor was pulled from a bound state to a dissociated state. The center of mass of the inhibitor was initially set at the origin of coordinates. To maintain the location of the 14-3-3σ protein during the SMD simulations, we restrained the z coordinates of several backbone carbon atoms of the 14-3-3σ protein which are far away from the binding pocket. Two compounds were equilibrated for 150000 steps for each segment without pulling forces and for 100000 steps with pulling forces.

### Potential of mean force (PMF) from steered molecular dynamics (SMD) simulation

For each segment, the two end states are denoted as A and B, respectively. The Gibbs free energy difference between state A and an intermediate state r (namely, the PMF) was given by the Brownian-dynamics fluctuation-dissipation theorem (BD-FDT)^38^,^66^ as


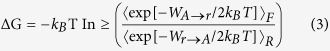


where *K*_*B*_ and *T* are the Boltzman constant and the absolute temperature, respectively. The brackets with subscript F and R represent the statistical average over the forward and reverse paths, respectively. 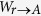 is the work done to the system along a forward path when the inhibitor is steered from A to r. 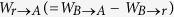 is the work done to the system along a reverse path when the inhibitor is steered from r to A.

## Additional Information

**How to cite this article**: Hu, G. *et al.* Revealing the binding modes and the unbinding of 14-3-3σ proteins and inhibitors by computational methods. *Sci. Rep.* **5**, 16481; doi: 10.1038/srep16481 (2015).

## Supplementary Material

Supplementary Information

## Figures and Tables

**Table 1:** Binding free energies calculated for nine compounds^a^.

Compound^b^	R1		R2		R3		R4		R5		R6		R7		R8		R9	
Items	Mean	σ^c^	Mean	σ^c^	Mean	σ^c^	Mean	σ^c^	Mean	σ^c^	Mean	σ^c^	Mean	σ^c^	Mean	σ^c^	Mean	σ^c^
ΔE_ele_	214.42	1.31	152.11	2.12	242.33	1.36	246.06	1.33	230.44	1.16	267.29	1.29	276.22	1.20	316.32	1.20	222.79	1.35
ΔE_vdw_	−13.98	0.23	−6.73	0.27	−15.21	0.23	−13.68	0.24	−12.73	0.22	−14.30	0.23	−14.45	0.23	−13.73	0.22	−11.82	0.23
ΔG_pol_	−247.44	1.12	−188.06	1.80	−268.85	1.15	−272.16	1.15	−256.08	1.03	−287.10	1.19	−295.70	1.10	−334.00	1.09	−254.20	1.19
ΔG_nonpol_	−2.53	0.01	−2.23	0.01	−2.53	0.01	−2.51	0.01	−2.63	0.01	−2.48	0.01	−2.76	0.01	−2.69	0.01	−2.59	0.01
ΔG_ele+pol_	−33.02	1.21	−35.95	1.96	−26.52	1.26	−26.10	1.24	−25.64	1.09	−19.81	1.24	−19.48	1.15	−17.68	1.15	−31.41	1.27
ΔG_vdw+nonpol_	−16.51	0.12	−8.96	0.14	−17.74	0.12	−16.19	0.12	−15.36	0.11	−16.78	0.12	−17.21	0.12	−16.42	0.11	−14.41	0.12
ΔH	−49.53	0.32	−44.91	0.42	−44.26	0.32	−42.29	0.31	−41.00	0.29	−36.59	0.24	−36.69	0.23	−34.10	0.23	−45.82	0.34
−ΔTS	18.99	0.45	19.85	0.34	20.99	0.37	19.90	0.48	20.04	0.44	20.33	0.45	21.04	0.38	21.37	0.32	19.81	0.46
ΔG_bind_	−30.54	0.55	−25.06	0.58	−23.27	0.44	−22.39	0.55	−20.96	0.48	−16.26	0.48	−15.65	0.37	−12.73	0.36	−26.01	0.57
ΔG_exp_^d^	−7.28		−6.62		−6.58		−6.27		−6.21		−6.17		−6.10		−5.19		null	

^a^All values are given in kcal/mol.

^b^The symbols of the energy terms are described in the section of the binding free energy calculations.

^c^Standard errors were calculated by *σ* = standard diviation/N^1/2^ 31,67.

^d^The experimental binding free energies were calculated according to the 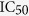 by 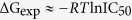.

**Table 2:** The hydrogen bonds of group one with protein in each compond^a^.

Donor	Arg60-Nh1		Arg60-NH2		Arg60-NH1		Arg-133NH1		Arg133-NH2		Arg-133NH1		Tyr134-OH	
Acceptor^b^	O1		O3		O3		O3		O2		O2		O2	
Items^c^	Occ	Dis	Occ	Dis	Occ	Dis	Occ	Dis	Occ	Dis	Occ	Dis	Occ	Dis
R1	99.8	2.84	100.0	2.76	96.6	3.00	100.0	2.76	100.0	2.79	80.2	3.23	100.0	2.61
R2	100.0	2.77	99.8	2.76	68.8	3.27	100.0	2.73	100.0	2.79	52.8	3.32	100.0	2.64
R3	99.8	2.86	100.0	2.77	86.6	3.10	99.8	2.76	100.0	2.78	75.2	3.25	99.8	2.64
R4	99.2	2.84	99.8	2.78	87.8	3.12	100.0	2.79	100.0	2.78	89.4	3.18	100.0	2.65
R5	100.0	2.77	99.8	2.79	67.4	3.16	100.0	2.80	100.0	2.76	81.2	3.20	100.0	2.65
R6	100.0	2.83	100.0	2.76	91.4	3.10	100.0	2.77	100.0	2.75	85.2	3.22	100.0	2.64
R7	100.0	2.83	100.0	2.74	92.4	3.14	100.0	2.78	100.0	2.76	87	3.21	100.0	2.64
R8	99.8	2.81	100.0	2.74	76.6	3.27	100.0	2.77	100.0	2.76	74	3.27	100.0	2.63
R9	97.8	2.87	100.0	2.77	84.8	3.13	100.0	2.76	100.0	2.78	65.8	3.27	100.0	2.66

^a^The hydrogen bonds are determined by the distance between the acceptor and donor atoms less than 3.5 Å and the angle of the acceptor and H -donor great than 120°.

^b^Atomic names of the phosphate group as the donor of hydrogen bond.

^c^Occ and Dis are the occupancy and distance of hydrogen bonds.

## References

[b1] Mitra S., Cheng K. W. & Mills G. B. Rab GTPases implicated in inherited and acquired disorders. Semin Cell Dev Biol 22, 57–68 (2011). 21147240 10.1016/j.semcdb.2010.12.005 PMC3395236

[b2] Jailkhani N., Chaudhri V. K. & Rao K. V. Regulatory cascades of protein phosphatases: implications for cancer treatment. Anticancer Agents Med Chem 11, 64–77 (2011). 21214508 10.2174/187152011794941253

[b3] Zhang Q. C. *et al.* Structure-based prediction of protein-protein interactions on a genome-wide scale. Nature 490, 556–560 (2012). 23023127 10.1038/nature11503 PMC3482288

[b4] Hermeking H. & Benzinger A. 14-3-3 proteins in cell cycle regulation. Semin Cancer Biol 16, 183–192 (2006). 16697662 10.1016/j.semcancer.2006.03.002

[b5] Fu H., Subramanian R. R. & Masters S. C. 14-3-3 proteins: structure, function, and regulation. Annu Rev Pharmacol Toxicol 40, 617–647 (2000). 10836149 10.1146/annurev.pharmtox.40.1.617

[b6] Powell D. W. *et al.* Proteomic identification of 14-3-3zeta as a mitogen-activated protein kinase-activated protein kinase 2 substrate: role in dimer formation and ligand binding. Mol Cell Biol 23, 5376–5387 (2003). 12861023 10.1128/MCB.23.15.5376-5387.2003 PMC165733

[b7] Aitken A. 14-3-3 proteins: a historic overview. Semin Cancer Biol 16, 162–172 (2006). 16678438 10.1016/j.semcancer.2006.03.005

[b8] Urano T. *et al.* Efp targets 14-3-3 sigma for proteolysis and promotes breast tumour growth. Nature 417, 871–875 (2002). 12075357 10.1038/nature00826

[b9] Li Z. *et al.* Determinants of 14-3-3sigma protein dimerization and function in drug and radiation resistance. J Biol Chem 288, 31447–31457 (2013). 24043626 10.1074/jbc.M113.467753 PMC3814741

[b10] Milroy L. G., Brunsveld L. & Ottmann C. Stabilization and inhibition of protein-protein interactions: the 14-3-3 case study. ACS Chem Biol 8, 27–35 (2013). 23210482 10.1021/cb300599t

[b11] Liu D. *et al.* Crystal structure of the zeta isoform of the 14-3-3 protein. Nature 376, 191–194 (1995). 7603574 10.1038/376191a0

[b12] Yang X. *et al.* Structural basis for protein-protein interactions in the 14-3-3 protein family. Proc Natl Acad Sci USA 103, 17237–17242 (2006). 17085597 10.1073/pnas.0605779103 PMC1859916

[b13] Rauh D. Chemical Biology—Current and Next Challenges. ACS Chemical Biology 8, 1–2 (2013). 23327350 10.1021/cb400013t

[b14] Kaiser M. & Ottmann C. The first small-molecule inhibitor of 14-3-3 s: modulating the master regulator. Chembiochem 11, 2085–2087 (2010). 20839267 10.1002/cbic.201000483

[b15] Wu H., Ge J. & Yao S. Q. Microarray-assisted high-throughput identification of a cell-permeable small-molecule binder of 14-3-3 proteins. Angew Chem Int Ed Engl 49, 6528–6532 (2010). 20677307 10.1002/anie.201003257

[b16] Corradi V. *et al.* Identification of the first non-peptidic small molecule inhibitor of the c-Abl/14-3-3 protein-protein interactions able to drive sensitive and Imatinib-resistant leukemia cells to apoptosis. Bioorg Med Chem Lett 20, 6133–6137 (2010). 20832303 10.1016/j.bmcl.2010.08.019

[b17] Zhao J. *et al.* Discovery and structural characterization of a small molecule 14-3-3 protein-protein interaction inhibitor. Proc Natl Acad Sci USA 108, 16212–16216 (2011). 21908710 10.1073/pnas.1100012108 PMC3182712

[b18] Roglin L., Thiel P., Kohlbacher O. & Ottmann C. Covalent attachment of pyridoxal-phosphate derivatives to 14-3-3 proteins. Proc Natl Acad Sci USA 109, E1051–E1053 (2012). 22532669 10.1073/pnas.1116592109 PMC3345018

[b19] Bier D. *et al.* Molecular tweezers modulate 14-3-3 protein-protein interactions. Nat Chem 5, 234–239 (2013). 23422566 10.1038/nchem.1570

[b20] Thiel P. *et al.* Virtual screening and experimental validation reveal novel small-molecule inhibitors of 14-3-3 protein-protein interactions. Chem Commun (Camb) 49, 8468–8470 (2013). 23939230 10.1039/c3cc44612c

[b21] Li W., Wang W. & Takada S. Energy landscape views for interplays among folding, binding, and allostery of calmodulin domains. Proc Natl Acad Sci USA 111, 10550–10555 (2014). 25002491 10.1073/pnas.1402768111 PMC4115553

[b22] Chen J., Wang J., Zhu W. & Li G. A computational analysis of binding modes and conformation changes of MDM2 induced by p53 and inhibitor bindings. J Comput Aided Mol Des 27, 965–974 (2013). 24264557 10.1007/s10822-013-9693-z

[b23] Hu G. & Wang J. Ligand selectivity of estrogen receptors by a molecular dynamics study. Eur J Med Chem 74, 726–735 (2014). 23694906 10.1016/j.ejmech.2013.04.049

[b24] Chen J. *et al.* Revealing origin of decrease in potency of darunavir and amprenavir against HIV-2 relative to HIV-1 protease by molecular dynamics simulations. Scientific reports 4, 6872 (2014). 25362963 10.1038/srep06872 PMC4217091

[b25] Shen J. *et al.* Discovery of potent ligands for estrogen receptor beta by structure-based virtual screening. Journal of medicinal chemistry 53, 5361–5365 (2010). 20553023 10.1021/jm100369g

[b26] Stoica I., Sadiq S. K. & Coveney P. V. Rapid and Accurate Prediction of Binding Free Energies for Saquinavir-Bound HIV-1 Proteases. J Am Chem Soc 130, 2639–2648 (2008). 18225901 10.1021/ja0779250

[b27] Hou T., Wang J., Li Y. & Wang W. Assessing the performance of the MM/PBSA and MM/GBSA methods. 1. The accuracy of binding free energy calculations based on molecular dynamics simulations. J Chem Inf Model 51, 69–82 (2011). 21117705 10.1021/ci100275a PMC3029230

[b28] Chen J., Wang X., Zhu T., Zhang Q. & Zhang J. Z. A Comparative Insight into Amprenavir Resistance of Mutations V32I, G48V, I50V, I54V, and I84V in HIV-1 Protease Based on Thermodynamic Integration and MM-PBSA Methods. J Chem Inf Model 55, 1903–1913 (2015). 26317593 10.1021/acs.jcim.5b00173

[b29] Deng Y. & Roux B. Computations of standard binding free energies with molecular dynamics simulations. J Phys Chem B 113, 2234–2246 (2009). 19146384 10.1021/jp807701h PMC3837708

[b30] Gohlke H., Kiel C. & Case D. A. Insights into protein-protein binding by binding free energy calculation and free energy decomposition for the Ras-Raf and Ras-RalGDS complexes. J Mol Biol 330, 891–913 (2003). 12850155 10.1016/s0022-2836(03)00610-7

[b31] Yang Y., Shen Y., Liu H. & Yao X. Molecular dynamics simulation and free energy calculation studies of the binding mechanism of allosteric inhibitors with p38alpha MAP kinase. J Chem Inf Model 51, 3235–3246 (2011). 22097958 10.1021/ci200159g

[b32] Lee M. C. & Duan Y. Distinguish protein decoys by using a scoring function based on a new AMBER force field, short molecular dynamics simulations, and the generalized born solvent model. Proteins 55, 620–634 (2004). 15103626 10.1002/prot.10470

[b33] Weiser J., Shenkin P. S. & Still W. C. Approximate atomic surfaces from linear combinations of pairwise overlaps (LCPO). J Comput Chem 20, 217–230 (1999).

[b34] Tsui V. & Case D. A. Theory and applications of the generalized Born solvation model in macromolecular simulations. Biopolymers 56, 275–291 (2000). 11754341 10.1002/1097-0282(2000)56:4<275::AID-BIP10024>3.0.CO;2-E

[b35] Grubmuller H., Heymann B. & Tavan P. Ligand binding: molecular mechanics calculation of the streptavidin-biotin rupture force. Science 271, 997–999 (1996). 8584939 10.1126/science.271.5251.997

[b36] Patel J. S., Berteotti A., Ronsisvalle S., Rocchia W. & Cavalli A. Steered Molecular Dynamics Simulations for Studying Protein–Ligand Interaction in Cyclin-Dependent Kinase 5. Journal of Chemical Information and Modeling 54, 470–480 (2014). 24437446 10.1021/ci4003574

[b37] Li M. S. & Mai B. K. Steered molecular dynamics—a promising tool for drug design. Curr. Bioinform. 7, 342 (2012).

[b38] Chen L. Y. Nonequilibrium fluctuation-dissipation theorem of Brownian dynamics. J Chem Phys 129, 144113 (2008). 19045140 10.1063/1.2992153 PMC2671654

[b39] Chen L. Y. Glycerol modulates water permeation through Escherichia coli aquaglyceroporin GlpF. Biochim Biophys Acta 1828, 1786–1793 (2013). 23506682 10.1016/j.bbamem.2013.03.008 PMC3761968

[b40] Chen L. Y. Exploring the free-energy landscapes of biological systems with steered molecular dynamics. Phys Chem Chem Phys 13, 6176–6183 (2011). 21359274 10.1039/c0cp02799e PMC3111135

[b41] Hu G. & Chen L. Y. In silico experiments of single-chain antibody fragment against drugs of abuse. Biophys Chem 153, 97–103 (2010). 21056529 10.1016/j.bpc.2010.10.008 PMC3006299

[b42] Allen T. W., Andersen O. S. & Roux B. Molecular dynamics—potential of mean force calculations as a tool for understanding ion permeation and selectivity in narrow channels. Biophys Chem 124, 251–267 (2006). 16781050 10.1016/j.bpc.2006.04.015

[b43] Colizzi F., Perozzo R., Scapozza L., Recanatini M. & Cavalli A. Single-molecule pulling simulations can discern active from inactive enzyme inhibitors. J Am Chem Soc 132, 7361–7371 (2010). 20462212 10.1021/ja100259r

[b44] Giorgino T. & De Fabritiis G. A High-Throughput Steered Molecular Dynamics Study on the Free Energy Profile of Ion Permeation through Gramicidin A. J. Chem. Theory Comput. 7, 1943 (2011). 10.1021/ct100707s 26596455

[b45] Mai B. K. & Li M. S. Neuraminidase inhibitor R-125489—a promising drug for treating influenza virus: steered molecular dynamics approach. Biochem Biophys Res Commun 410, 688–691 (2011). 21693105 10.1016/j.bbrc.2011.06.057

[b46] Henin J., Tajkhorshid E., Schulten K. & Chipot C. Diffusion of glycerol through Escherichia coli aquaglyceroporin GlpF. Biophys J 94, 832–839 (2008). 17921212 10.1529/biophysj.107.115105 PMC2186255

[b47] Mai B. K., Viet M. H. & Li M. S. Top leads for swine influenza A/H1N1 virus revealed by steered molecular dynamics approach. J Chem Inf Model 50, 2236–2247 (2010). 21090736 10.1021/ci100346s

[b48] Nicolini P., Frezzato D., Gellini C., Bizzarri M. & Chelli R. Toward quantitative estimates of binding affinities for protein-ligand systems involving large inhibitor compounds: a steered molecular dynamics simulation route. J Comput Chem 34, 1561–1576 (2013). 23620471 10.1002/jcc.23286

[b49] Wilker E. W., Grant R. A., Artim S. C. & Yaffe M. B. A structural basis for 14-3-3sigma functional specificity. J Biol Chem 280, 18891–18898 (2005). 15731107 10.1074/jbc.M500982200

[b50] Hu G., Li H., Liu J.-Y. & Wang J. Insight into Conformational Change for 14-3-3σ Protein by Molecular Dynamics Simulation. Int J Mol Sci 15, 2794–2810 (2014). 24552877 10.3390/ijms15022794 PMC3958882

[b51] Unni S. *et al.* Web servers and services for electrostatics calculations with APBS and PDB2PQR. J Comput Chem 32, 1488–1491 (2011). 21425296 10.1002/jcc.21720 PMC3062090

[b52] Humphrey W., Dalke A. & Schulten K. VMD: visual molecular dynamics. Journal of molecular graphics 14, 33–38, 27-38 (1996). 8744570 10.1016/0263-7855(96)00018-5

[b53] Obsil T. & Obsilova V. Structural basis of 14-3-3 protein functions. Semin Cell Dev Biol 22, 663–672 (2011). 21920446 10.1016/j.semcdb.2011.09.001

[b54] Case D. A. *et al.* The Amber biomolecular simulation programs. J Comput Chem 26, 1668–1688 (2005). 16200636 10.1002/jcc.20290 PMC1989667

[b55] Schumacher B., Skwarczynska M., Rose R. & Ottmann C. Structure of a 14-3-3sigma-YAP phosphopeptide complex at 1.15 A resolution. Acta Crystallogr Sect F Struct Biol Cryst Commun 66, 978–984 (2010). 10.1107/S1744309110025479 PMC2935210 20823509

[b56] Yong D. *et al.* A point-charge force field for molecular mechanics simulations of proteins based on condensed-phase quantum mechanical calculations. J Comput Chem 24, 1999–2012 (2003). 14531054 10.1002/jcc.10349

[b57] Frisch M. J. *et al.* Gaussian 03, Revision C.02. Gaussian, Inc., Wallingford CT (2004).

[b58] Case D. A. *et al.* AMBER 10. University of California, San Francisco (2008).

[b59] Jorgensen W. L., Chandrasekhar J., Madura J. D., Impey R. W. & Klein M. L. Comparison of Simple Potential Functions for Simulating Liquid Water. J Comput Phys 79, 926–935 (1983).

[b60] Darden T., York D. & Pedersen L. Particle Mesh Ewald -and.Log(N) Method for Ewald Sums in Large Systems. J Comput Phys 98, 10089–10092 (1993).

[b61] Ryckaert J. P., Ciccotti G. & Berendsen H. J. C. Numerical-Integration of Cartesian Equations of Motion of a System with Constraints—Molecular-Dynamics of N-Alkanes. J Comput Phys 23, 327–341 (1977).

[b62] Michel F. S., Arthur J. O. & Jean-Claude S. Reduced surface: An efficient way to compute molecular surfaces. Biopolymers 38, 305–320 (1996). 8906967 10.1002/(SICI)1097-0282(199603)38:3%3C305::AID-BIP4%3E3.0.CO;2-Y

[b63] Onufriev A., Bashford D. & Case D. A. Exploring protein native states and large-scale conformational changes with a modified generalized born model. Proteins 55, 383–394 (2004). 15048829 10.1002/prot.20033

[b64] Case D. A. Normal mode analysis of protein dynamics. Curr Opin Struc Biol 4, 285–290 (1994).

[b65] Jiang W. *et al.* High-performance scalable molecular dynamics simulations of a polarizable force field based on classical Drude oscillators in NAMD. J Phys Chem Lett 2, 87–92 (2011). 21572567 10.1021/jz101461d PMC3092300

[b66] Chen L. Y. Hybrid Steered Molecular Dynamics Approach to Computing Absolute Binding Free Energy of Ligand-Protein Complexes: A Brute Force Approach That Is Fast and Accurate. Journal of chemical theory and computation 11, 1928–1938 (2015). 25937822 10.1021/ct501162f PMC4411208

[b67] Genheden S. & Ryde U. How to obtain statistically converged MM/GBSA results. J Comput Chem 31, 837–846 (2010). 19598265 10.1002/jcc.21366

