# Supplementary material for: Revealing the binding modes and the unbinding of 14-3-3σ proteins and inhibitors by computational methods
Source: Sci Rep. 2015 Nov 16;5:16481. doi: 10.1038/srep16481 (PMC4644958; doi:10.1038/srep16481)
Supplement: Supplementary Information [file srep16481-s1.pdf]

Revealing the binding modes and the unbinding of 14-3-3σ proteins and inhibitors by computational methods

Guodong Hu[1], Zanxia Cao[1], Shicai Xu[1], Wei Wang[2,*], Jihua Wang[1,*]

[1] Shandong Provincial Key Laboratory of Functional Macromolecular Biophysics and College of Physics and Electronic Information, Dezhou University, Dezhou, 253023, China;

[2] National Laboratory of Solid State Microstructure and Department of Physics, Nanjing University, Nanjing, 210093, China

Authors to whom correspondence should be addressed; E-Mail: wangwei@nju.edu.cn; (W. W.) jhw25336@126.com (J. W.); Tel.: +86-534-8985879; Fax: +86-534-8985884.

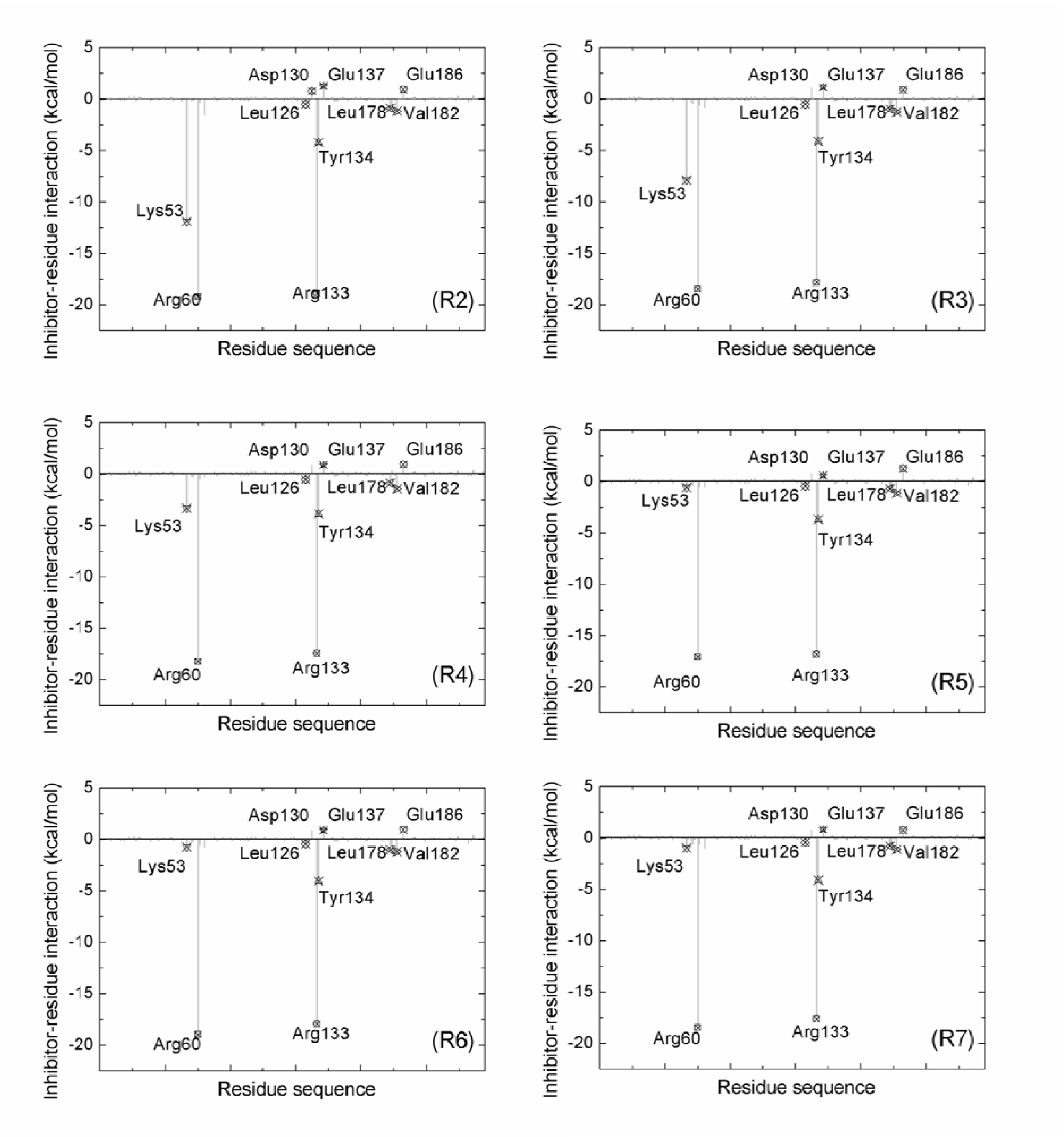

Figure S1. Decomposition of on a per-residue basis for the compounds R2- R7.

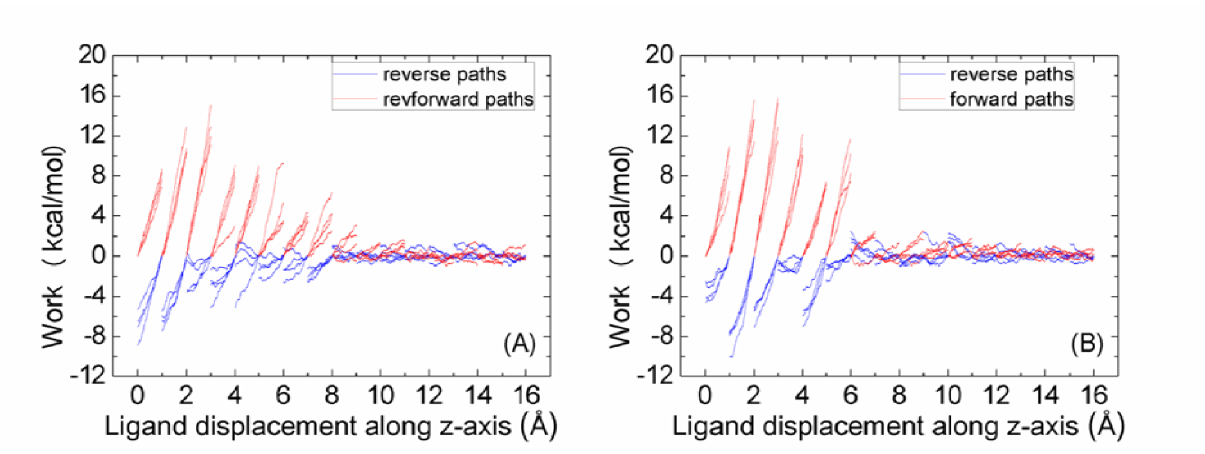

Figure S2. Plots A and B are the work done along the forward and reverse pulling paths for compound R1 and R8, respectively. In the forward direction, the atoms of a inhibitor were pulled away from the binding site. In the reverse direction, the atoms of a inhibitor were pulled back to the binding site.
